# A vision for reinvigorating global mental health

**DOI:** 10.1371/journal.pgph.0003034

**Published:** 2024-04-02

**Authors:** Anna Chiumento, Angus MacBeth, Rosie Stenhouse, Lotte Segal, Ian Harper, Sumeet Jain

**Affiliations:** 1 Social Work, School of Social and Political Sciences, University of Edinburgh, Edinburgh, United Kingdom; 2 Clinical Psychology, School of Health in Social Sciences, University of Edinburgh, Edinburgh, United Kingdom; 3 Nursing Studies, School of Health in Social Sciences, University of Edinburgh, Edinburgh, United Kingdom; 4 Social Anthropology, School of Social and Political Sciences, University of Edinburgh, Edinburgh, United Kingdom; University College London, UNITED KINGDOM

## Introduction

Across individuals, communities, and ecosystems, unequal ways of living mean different experiences and expressions of wellbeing and distress. What would it mean for Global Mental Health (GMH) to start with these disparities in lived-experience, radically, rather than take them as stumbling blocks to unite under a broad GMH umbrella? This question builds on a tradition of critically appraising the GMH field (see e.g. [1]), seeking a re-shaping of GMH around a shared vision deeply rooted in relationality, equity, and care.

### *Inter*disciplinary conversations

To illustrate our conversations, we present a thought experiment drawn from our work which foregrounds how an *inter*disciplinary position leads to divergent responses to experiences of wellbeing or distress than from disciplinary silos:

*An older woman from a marginalized community discriminated against by the majority ethnic group starts hearing voices. Her family are experiencing poverty and housing insecurity, partly driven by costs relating to her sons’ marriages. The older womans’ experiences of hearing voices leads to behaviours including running away and shouting at others. Her experiences and behaviours lead to conflict within the family over how to respond. (adapted with permission from [2*])

Readers of different disciplinary orientations will view this case differently. A Psychiatrist may prioritise the hearing of voices, interpreting this via a biomedical diagnostic frame to recommend anti-psychotic medication. A Clinical Psychologist may formulate the interplay of the presenting problem, predisposing factors, and current context with an evidence-based psychological intervention. A Cultural Psychiatrist may unpack the presenting symptomatology in terms of culturally specific idioms. Or a Sociologist may seek to understand the wellbeing impacts of structural and social injustices, interpreting these through axes of gender, identity, and cultural norms to view the hearing of voices as an expression of distress or resistance.

Surfacing the ways that our disciplinary lenses direct our attention enables exploring the intersections and tensions in how we view, interpret, and respond to experiences of distress. We open-up the diverse, and at times conflicting, normative ends of disciplinary and professional engagements with complex social lives that are mindful of the contributions, and responsibilities, we hold in promoting a given response. Ultimately, we embrace an expansive and ontologically, epistemologically, and ethically aware *inter*disciplinary approach in pursuit of global solidarities, social and environmental justice, and the common good [3].

### Valuing the diversity of knowledge

Starting with disparities problematises the structural conditions of GMH knowledge production initiated by primarily Global North-set research priorities shaped by clinical orientations towards mental health [4]. Funding allocation is accompanied by due diligence that entrenches power hegemonies, as Global North researchers hold senior positions whilst Global South researchers occupy co-investigator or precarious fieldworker roles, despite frequently being responsible for day-to-day research labour in which intense ethical issues arise [5]. Global North/South inequities act to reproduce structural inequalities, shaping access to academic resources [6] compounded by the hegemony of English [7]. This systematic exclusion of diverse ontologies, epistemologies, and their (written) expression, limits the normative horizon for what mental health care might entail (see e.g. [8]). Embracing a commitment to contextualising knowledges, we also critically interrogate whether the notion of culture necessarily offers an adequate response to capturing the diversity of experiences of distress and wellbeing. In saying this, we acknowledge the importance of a sensibility to the institutionalised cultures of knowledge production which systematically include some voices whilst excluding others.

This structural context has concrete implications: as educators we benefit from student diversity which enriches debate, revisiting tensions around what is recognised and revered as knowledge, the theories and methods underpinning its production; alongside the glocal creation of so-called evidence-based practice, treatment, and care. Students from majority countries may struggle to see their indigenous experiences reflected, leaving them feeling unrepresented and marginalised. Approaching this requires acknowledging structural inequalities, whilst not inadvertently reproducing these by reverting to English-language resources due to their availability (thus implying authority).

Globalised and diverse learning spaces offer important sites for problematising the GMH field, and for valuing the diversity of worldviews and forms of knowledge. What does a truly global and *inter*disciplinary GMH curriculum inclusive of experiences of mental health and wellbeing and its ways of knowing, expressing, and being entail? How can GMH research, practice, and teaching value reciprocal care and solidarities with globalised others [9]?

### Valuing the local and the social: The idea of lived-experience

In foregrounding lived experience, we recognise the ethical tensions embedded within this discourse, and its translation into practice (see e.g. [10]). In our conceptualisation, taking the local and the social seriously requires “ontological dignity” [11], allowing lived-experiences space to breathe and be heard, rather than being overdetermined via pre-defined categories of biomedical or social organisation [12]. What impact would it have on our research, practice, and teaching to meaningfully and purposefully attend to lives lived in diverse geographical, linguistic, social, and relational contexts? These are the constitutive environments in which wellbeing, emotional expression, idioms of distress, and health behaviours are enacted on a day-to-day basis; and into which (global) mental health interventions are implemented. Our positioning values integrating different forms of evidence about *how* and *why* certain practices can impact on mental health and wellbeing in varied and complex ways, attending to practice-based evidence [13] that critically interrogates complexity.

Meaningfully calibrating how diverse experiences of mental wellbeing and distress can be understood requires us to reconfigure our current systems of thinking and knowing. The temptation is to suggest a better approach which avoids the pitfalls we have recognised, yet that will not facilitate re-shaping the GMH field. Instead, we support engagement with the complexity of mental health as relationally and socially situated. Working with(in) complexity prioritises core principles [14]: firstly, centring lived experience as expertise. From this position, researchers must retain humility towards their position, being prepared to embrace a facilitator role focussed on best serving the group and challenging power relations to produce equitable knowledge structures. Alongside this the research is consciously embraces being non-directive and does not have a preconceived idea of the endpoint, to being attuned to the processes through which knowledge(s) emerge(s). This encourages the researcher to sit with the complexities, and discomforts, that arise in a reconceptualization of how we define, approach, and conduct research.

We reflect on GMH calls to implement and scale-up based on existing, partial, understandings of mental health. We propose embracing the ‘slow research movement’ which entails: “…working with an ethic or set of values and strategies that valorise different things from the emergent norms” [15, p.180]. We call for stepping back from and interrogating evidence-based medicine hierarchies and the processes and products that this generates, to instead pause and “think openly and conversationally” [15, p.179] about which approaches best serve the goals of ethically-oriented, situational, and relational GMH research and practice.

Grounded in these reflections, we advocate for a GMH field founded on principles of equity, relationality, and care; one that shifts knowledge production and power across disciplinary silos, legacies of academic (and clinical) power, and that fully engages with the rich complexity of lived experience. Working in this way necessarily problematizes whether the current epistemic and structural foundations of GMH can achieve the intended normative ends of mental health equity. We propose that to achieve this, there is a need to critically hold multiple concurrent sources of disciplinary evidence and to integrate the plurality of forms of data and lived experience that are reflective of the complexity inherent to unequal lives lived.

## References

[1] Kirmayer L, Pedersen D. Toward a new architecture for global mental health. Transcultural Psychiatry. 2014;51(6):759–76. doi: 10.1177/1363461514557202 25358524

[2] Jadhav S, Jain S. Clinical appeal of cultural formulations in rural mental health: a manual. In: Chavan BS, Gupta N, Arun P, Sidana A, Jadhav S, editors. Community mental health in India New Delhi: Jaypee; 2012. p. 560–5.

[3] Richie C. Introduction. Global Bioethics. 2022;33(1):1–3.

[4] Pollitt A, Cochrane G, Kirtley A, Krapels J, Larivière V, Lichten CA, et al. Project Ecosystem: A global map of mental health research funding. Santa Monica, CA: RAND Corporation; 2016. 10.7249/rhq-06-01-11 PMC5158275 28083439

[5] Molyneux S, Kamuya D, Madiega PA, Chantler T, Angwenyi V, Geissler PW. Field workers at the interface. Dev World Bioeth. 2013;13(1):ii–iv. doi: 10.1111/dewb.12027 23521824 PMC3662993

[6] Zhang M, Doi L, Awua J, Asare H, Stenhouse R. Challenges and possible solutions for accessing scholarly literature among medical and nursing professionals and students in low-and-middle income countries: A systematic review. Nurse Education Today. 2023;123:105737. doi: 10.1016/j.nedt.2023.105737 36753870

[7] Abimbola S. The foreign gaze: authorship in academic global health. BMJ Global Health. 2019;4(5):e002068. doi: 10.1136/bmjgh-2019-002068 31750005 PMC6830280

[8] Mills C, Davar B. A Local Critique of Global Mental Health. In: Grech S, Soldatic K, editors. Disability in the Global South: The Critical Handbook. Cham: Springer International Publishing; 2016. p. 437–51.

[9] Abimbola S, Asthana S, Montenegro C, Guinto RR, Jumbam DT, Louskieter L, et al. Addressing power asymmetries in global health: Imperatives in the wake of the COVID-19 pandemic. PLOS Medicine. 2021;18(4):e1003604. doi: 10.1371/journal.pmed.1003604 33886540 PMC8101997

[10] Joseph A. Constituting “lived experience” discourses in mental health: the ethics of racialized identification/representation and the erasure of intergeneration colonial violence. Journal of Ethics in Mental Health. 2019;10(Special Theme Issue VI: Disordering Social Inclusion).

[11] Morizot B. Postscript: Gathering up the Knowledge which has Fallen from the Nest. In: Despret V, Morrison H, editors. Living as a bird Vinciane Despret; translated by Helen Morrison. Cambridge: Polity Press; 2022. p. 85–7.

[12] Biehl JoG, Petryna A. When people come first: critical studies in global health / edited by João Biehl & Adriana Petryna. Princeton, New Jersey;: Princeton University Press; 2013.

[13] Kienzler H. Mental Health System Reform in Contexts of Humanitarian Emergencies: Toward a Theory of “Practice-Based Evidence”. Culture, Medicine, and Psychiatry. 2019;43(4):636–62. 31729689 10.1007/s11013-019-09641-w

[14] Kahane A. Facilitating Breakthrough: How to Remove Obstacles, Bridge Differences, and Move Forward Together. Beaverton: Ringgold, Inc; 2021.

[15] Adams V, Burke NJ, Whitmarsh I. Slow Research: Thoughts for a Movement in Global Health. Medical Anthropology. 2014;33(3):179–97. doi: 10.1080/01459740.2013.858335 24761973

